# Evaluation of viability, developmental competence, and apoptosis-related transcripts during *in vivo* post-ovulatory oocyte aging in zebrafish *Danio rerio* (Hamilton, 1822)

**DOI:** 10.3389/fvets.2024.1389070

**Published:** 2024-06-17

**Authors:** Essaikiammal Sodalai Muthu Konar, Knut Mai, Sebastian Brachs, Swapnil Gorakh Waghmare, Azadeh Mohagheghi Samarin, Tomas Policar, Azin Mohagheghi Samarin

**Affiliations:** ^1^Research Institute of Fish Culture and Hydrobiology, South Bohemian Research Center of Aquaculture and Biodiversity of Hydrocenoses, Faculty of Fisheries and Protection of Waters, University of South Bohemia in Ceske Budejovice, Vodnany, Czechia; ^2^Department of Endocrinology and Metabolism, Charité – Universitätsmedizin Berlin, Corporate Member of Freie Universität Berlin and Humboldt-Universität zu Berlin, Berlin, Germany; ^3^DZHK (German Centre for Cardiovascular Research), Partner Site Berlin, Berlin, Germany

**Keywords:** apoptosis, cell death, fertilization, membrane integrity, trypan blue, zebrafish

## Abstract

**Introduction:**

Post-ovulatory aging is a time-dependent deterioration of ovulated oocytes and a major limiting factor reducing the fitness of offspring. This process may lead to the activation of cell death pathways like apoptosis in oocytes.

**Methodology:**

We evaluated oocyte membrane integrity, egg developmental competency, and mRNA abundance of apoptosis-related genes by RT-qPCR. Oocytes from zebrafish *Danio rerio* were retained *in vivo* at 28.5°C for 24 h post-ovulation (HPO). Viability was assessed using trypan blue (TB) staining. The consequences of in vivo oocyte aging on the developmental competence of progeny were determined by the embryo survival at 24 h post fertilization, hatching, and larval malformation rates.

**Results:**

The fertilization, oocyte viability, and hatching rates were 91, 97, and 65% at 0 HPO and dropped to 62, 90, and 22% at 4 HPO, respectively. The fertilizing ability was reduced to 2% at 8 HPO, while 72% of oocytes had still intact plasma membranes. Among the apoptotic genes *bcl-2* (b-cell lymphoma 2), *bada* (bcl2-associated agonist of cell death a), *cathepsin D, cathepsin Z, caspase 6a, caspase 7, caspase 8, caspase 9, apaf1, tp53* (tumor protein p53), *cdk1* (cyclin-dependent kinase 1) studied, mRNA abundance of anti-apoptotic *bcl-2* decreased and pro-apoptotic *cathepsin D* increased at 24 HPO. Furthermore, *tp53* and *cdk1* mRNA transcripts decreased at 24 HPO compared to 0 HPO.

**Discussion:**

Thus, TB staining did not detect the loss of oocyte competency if caused by aging. TB staining, however, could be used as a simple and rapid method to evaluate the quality of zebrafish oocytes before fertilization. Taken together, our results indicate the activation of cell death pathways in the advanced stages of oocyte aging in zebrafish.

## 1 Introduction

The fertilization rate and subsequent normal embryo and larval developmental stages can be used to define the quality of gametes (1). A delay in fertilization of ovulated oocytes leads to post-ovulatory aging or over-ripeness. Post-ovulatory aging is a factor that adversely affects the quality of oocytes and is complicated to manage. This aging process causes a loss of developmental competence of the egg, as well as deterioration, and may lead to oocyte death. Based on previous studies using murine, porcine and bovine models, oxidative stress (2, 3), mitochondrial dysfunction (4–6), degradation of maternal RNAs (7, 8), loss of survival factors (9, 10), epigenetic modifications (11, 12), and apoptosis (13–15) are probably involved in this process of oocyte aging. Mitochondrial microRNAs for cell death and signal transduction, stress response and DNA damage, RNA degradation, as well as energy and transcription regulation, were downregulated in rainbow trout (*Oncorhynchus mykiss*) due to post-ovulatory aging (16). However, oxidative stress does presumably not initiate oocyte aging in common carp (*Cyprinus carpio*) and goldfish (*Carassius auratus*) (17, 18). Histone acetylation pattern at histone H4 altered during the oocyte aging in common carp (19) indicating the involvement of epigenetic regulation. The first report of upregulation of apoptotic genes in aged oocytes from common carp speculated about the involvement of apoptosis in the degenerative process of fish oocytes (17).

The optimal time window for fertilization after ovulation highly varies among species and environmental conditions like temperature and storage (20). Several studies have been carried out to understand the *in vitro* and *in vivo* oocyte aging process using different model species. For example, the fertilization rate of *in vivo* aged curimata (*Prochilodus marggravii*) oocytes declined more prominently compared to *in vitro* aged at 26°C (21). The successful *in vitro* storage of Caspian brown trout (*Salmo trutta caspius*) oocytes was for 2 days at 2–3°C (22), whereas *in vivo* storage was for 30 days even at a higher temperature of 7°C (23). The most suitable duration for *in vitro* storage of rainbow trout oocytes was 9 days (24), and for *in vivo* storage was 30 days even at the same temperature, i.e., 2°C (25). The *in vivo* storage of northern pike (*Esox lucius*) oocytes retained the maximum fertilizing ability till 4 days post ovulation, whereas *in vitro* aging of 24 h resulted in a low fertilization rate at 10°C (26). In this study, we established a 24-h *in vivo* oocyte aging of zebrafish (*Danio rerio*) for the first time, as *in vivo* studies are important to understand the fate of oocytes retained inside the fish body after ovulation.

The underlying mechanisms of the degeneration process of post-ovulatory aged oocytes in fish remain elusive. Therefore, in this study, we used zebrafish as the experimental animal to investigate the decrease of trypan blue (TB) -based viability during *in vivo* aging of zebrafish oocytes and their quality before fertilization. Furthermore, the developmental competence was analyzed by estimating fertilization capability, embryo survival, hatching, and larval malformation rates after fertilization. Moreover, we analyzed the mRNA abundance of the pro-survival factor *bcl-2* (b-cell lymphoma 2), cell cycle regulator *cdk1* (cyclin-dependent kinase 1), and the main pro-apoptotic protein *bada* (*bcl2*-associated agonist of cell death a) during oocyte aging. The pro-apoptotic regulators like *cathepsin D* and *cathepsin Z*, *tp53* (tumor protein p53), and *apaf1* (apoptosis peptidase activating factor 1) mRNA abundance were also investigated. In addition, *caspase 6a*, *caspase 7*, *caspase 8,* and *caspase 9* were also studied. The successful *in vivo* storage of the zebrafish AB strain oocytes was identified.

## 2 Materials and methods

### 2.1 Animal maintenance

The zebrafish (AB strain) used in this study were initially obtained from the European Zebrafish Resource Centre (Karlsruhe, Germany) and later donated to the Faculty of Fisheries and Protection of Waters, the University of South Bohemia, Vodnany, Czech Republic. The fish were maintained in the ZebTec Active Blue rearing system (Techniplast, UK) under optimal photoperiod (14:10 h for light:dark cycle) and temperature (28.5°C). The broodstock was fed an adequate amount of Skretting M-300 Gemma Micro (Skretting, UK), according to the manufacturers’ recommendation. The females were bred at two-week intervals to maintain a fresh reproductive cycle. For this experiment, approximately 12-month-old fish were used. The males and females were prepared for gamete Research Topic based on the protocol by Westerfield (27). Briefly, males and females were separated overnight in 1 L spawning tanks by a glass partition in the 2:1 ratio. The spawning was induced by the light on the subsequent morning.

### 2.2 Oocyte sampling

Ovulation was detected by observing the release of a few eggs by the female fish. After that, the females were anaesthetized with 0.05% tricaine methanesulfonate (methyl-aminobenzoate, MS222). The hand-stripping method was followed for the Research Topic of oocytes. A smidgen of oocytes (approx. 150 oocytes) was stripped from each of the six experimental females and considered as the fresh group or 0 h post-ovulation (HPO). The remaining oocytes were retained in the fish body for *in vivo* aging at 28.5°C. The cycle of collecting oocytes was continued at 2, 4, 8, and 24 HPO. Six individual females were used to strip at 0, 2 and 24 HPO, and another set of six females were used to strip at 0, 4 and 8 HPO since stripping the same females five times is unfeasible. The oocytes collected at each time point were divided into two different 35 mm petri dishes to assess viability loss using TB and for *in vitro* fertilization. The oocyte samples from 0, 8, and 24 HPOs were immediately frozen in liquid nitrogen and stored at −80°C till further use for total RNA extraction.

### 2.3 Oocyte membrane integrity assessment

Trypan blue dye exclusion test was used to analyze the oocytes’ membrane integrity. The stock solution of 0.2% trypan blue (Sigma-Aldrich, Missouri, United States) was prepared in 1X Phosphate Buffered Saline (PBS, Sigma-Aldrich). 30–60 oocytes were stained with TB staining solution (0.1% in PBS) for 2 min at room temperature in a 35 mm petri dish immediately after stripping. Following staining, the oocytes were rinsed with PBS before visualization. TB stain, a large negatively charged molecule, is impermeable to cells with an intact plasma membrane and permeable to a cell with a disrupted membrane and enters the cytoplasm, making non-viable cells dark blue (28). The total and the viable number of oocytes were counted under a stereo microscope (Olympus SZX16, Olympus Life Science, Japan). The number of viable oocytes to the total number of oocytes used for staining was used to calculate the viability rates.

### 2.4 Artificial insemination

The males were anaesthetized in MS222 (0.05%), and milt was collected from them in the immobilizing solution (Kurokura 180) (29). While stripping the male fish, a 2.5 μL pipette was used to collect the milt simultaneously. The milt collected from 5 males were pooled together in 50 μL of Kurokura 180 solution. The milt was pooled to eliminate the effect of individual male sperm quality and to have an equal volume of sperm for fertilization of all females. The motility of pooled sperm was analyzed using an Olympus CX31 light microscope (Olympus Life Science, Japan) before fertilization, according to Fauvel et al. (30). The pooled milt showed >75% of active spermatozoa movement. The oocytes stripped at 0, 2, and 24 HPO from six individual females, and 0, 4, and 8 HPO from another set of six females were fertilized immediately. Fertilization was carried out by adding 200 μL of water and 10 μL of sperm to 50–90 oocytes, as the number varied at different time points of aging in a 35 mm glass petri plate. The eggs were thereafter transferred into a Pyrex® Petri plate (9 cm in diameter, Germany) (31). After 90 min of incubation at 28.5°C, the fertilization rate was assessed by examining the cleavage pattern of embryos. The eggs showing cleavage were counted under a Nikon SMZ745T stereo microscope (Nikon, Japan). The fertilization rate was defined by the number of eggs showing cleavage to the total number of oocytes inseminated.

### 2.5 Assessment of egg developmental indices

Embryo survival, mortality, hatching, and malformation rates were calculated to determine the developmental competence of differently aged oocytes, according to Waghmare et al. (31). Briefly, the fertilized eggs were re-examined after 24 h of fertilization. The number of viable embryos after 24 h of fertilization to the total number of inseminated oocytes was used to determine the embryo survival rate. The successful development of fertilized eggs was examined by calculating embryo mortality (between 24 h and the hatching period) and hatching (48-72 h after fertilization) rates. The embryo mortality rate was determined by counting the viable embryos between 24 h and the hatching period to the total number of 24 h survived embryos. The number of hatched larvae to the total number of inseminated eggs was used to determine the hatching rate. After hatching, the larvae were observed under the Nikon SMZ745T stereomicroscope (Nikon, Japan) for the body shape. The larvae with body shape curved, bent, or curled were counted (72 h after fertilization) to the total number of hatched larvae for malformation rate.

### 2.6 RNA isolation and cDNA synthesis

Total RNA was isolated from 10 mg of oocytes sampled at 0, 8, and 24 HPOs from four individual females by the Trizol (Invitrogen, United States) method. The RNA concentration and quality were assessed using Nanodrop 2000 (Thermo Scientific, United States). The isolated RNA was treated with DNAse I, Amplification Grade (Invitrogen, United States) to digest residual DNA. Maxima First Strand cDNA Synthesis Kit (Thermo Scientific™, United States) was used for reverse transcription of 1,000 ng of RNA to first strand cDNA according to the manufacturer’s instructions. Reverse transcription was carried out by incubating samples for 10 min at 25°C followed by 15 min at 50°C and final termination at 85°C for 5 min.

### 2.7 Quantitative expression analysis

Real-time quantitative expression analysis was performed to determine the apoptosis-related gene (*bcl-2*, *bada*, *cathepsin D*, *cathepsin Z*, *caspase 6a*, *caspase 7*, *caspase 8*, *caspase 9*, *apaf1*, *p53*, *cdk1*) expression using LightCycler 480 (Roche Applied Science, Germany). The primers of targeted genes were designed by Primer3web (https://primer3.ut.ee/) and were provided by Eurofins Genomics, Germany (Table 1). The reaction mixture contained 2 μL of diluted cDNA (1:10), 1 μL forward and reverse primers (500 nM), 2 μL of nuclease-free water, and 5 μL PowerUp™ SYBR™ Green Master Mix (Thermo scientific™, United States). 18S ribosomal RNA (*18 s*), beta-actin (*βactin*), and elongation factor 1 alpha (*ef1α*) were tested as reference genes. Among those, 18S was found to be the most stable reference gene to normalize the expression of selected genes. All data were presented as mean ± SD from three samples with three parallel repetitions, and all the primers were validated by melt curve analysis. The relative expression levels were analyzed by the 2^-ΔΔCt^ method (32).

**Table 1:** primer sequence of selected genes for quantitative expression analysis.

Gene	Forward primer (5′-3′)	Reverse primer (5′-3′)	Genbank accession no
*bcl2*	GTCGAGTGTGTGGAGAAGGA	CCGCTGCATCTTTTCCAAAG	NM_131807.1
*bada*	ACCTCGCATGACCATCAAGA	AGATTCCGAATAGAGCCGCA	XM_005161364.4
*cathepsin D (ctsd)*	CCTGAAATACAACCTGGGCT	TGAAGGTCTGGACAGGAGTG	NM_131710.2
*cathepsin Z (ctsz)*	GCACTACACGCAACCAACAT	CCACAGTCAATCACGTTCTGG	NM_001006043.1
*caspase 6a*	ATGTCGTAGTCTTGTGGGCA	CCATCGGAGTCACAGGATCA	NM_001020497.1
*caspase 7*	ACCATGACCTCGCTCTTCAA	TATTGTGTCATTGGGCGGTC	NM_001020607.1
*caspase 8*	ATCTTCCAAGGGCAAAGCTG	GCCCAAGCCTCTGTTGTTTT	NM_131510.2
*caspase 9*	TCATCGCCCTCCTGTCATTT	AGGCTTTCAGGTCTCAGTGG	NM_001007404.2
*p53*	CTCTCCCACCAACATCCACT	TGCCAGCTGACAGAAGAGTT	U60804.1
*apaf-1*	CACAAACTCCCAGAACAGGC	AATGCGCTTGAACTGCTTCT	NM_131608.1
*cdk1*	CATCTTTGCTGAACTCGCCA	AGAGACTCAACATCTGGCCA	NM_212564.2
*βactin*	TAGTCATTCCAGAAGCGTTTACC	TACAGAGACACCCTGGCTTACAT	AF057040.1
*18S rRNA*	GAATTCCCAGTAAGCGCAGG	GATCCGAGGACCTCACTGAG	BX296557.35
*ef1α*	ACAGCTGATCGTTGGAGTCA	GTATGCGCTGACTTCCTTGG	L23807.1

### 2.8 Data analysis

The statistical evaluation and graph generation were performed using GraphPad Prism 9.4.1 (GraphPad Software, San Diego, CA, United States). The viability, fertilization, egg development indices, and relative gene expression were analyzed for variance by one-way ANOVA followed by Tukey’s multiple comparisons tests for *post hoc* comparisons. Viability and fertilization rates between the high- and low-quality oocytes were analyzed by Mann Whitney test as data were not normally distributed. Statistical significance was considered with *p ≤ 0.05, **p ≤ 0.01, ***p ≤ 0.001, and, ****p ≤ 0.0001.

### 2.9 Compliance with ethical standards

All methodological protocols, experimental manipulations, and sampling procedures used in the present study were approved by the expert committee of the Institutional Animal Care and Use Committee of the University of South Bohemia, Czech Republic. The co-authors of this study deal with the manipulation and artificial reproduction of fish and hold certificates authorizing them to work with laboratory animals according to section 15d paragraph 3 of Act no. 246/1992 Coll. For the purposes of stripping gametes, fish were anesthetized with 0.05% tricaine methanesulfonate (MS-222; Sigma-Aldrich, United States) to ensure their welfare and minimize any associated stress.

## 3 Results

### 3.1 Viability loss and fertilization during *in vivo* oocyte aging

**Figure 1:**
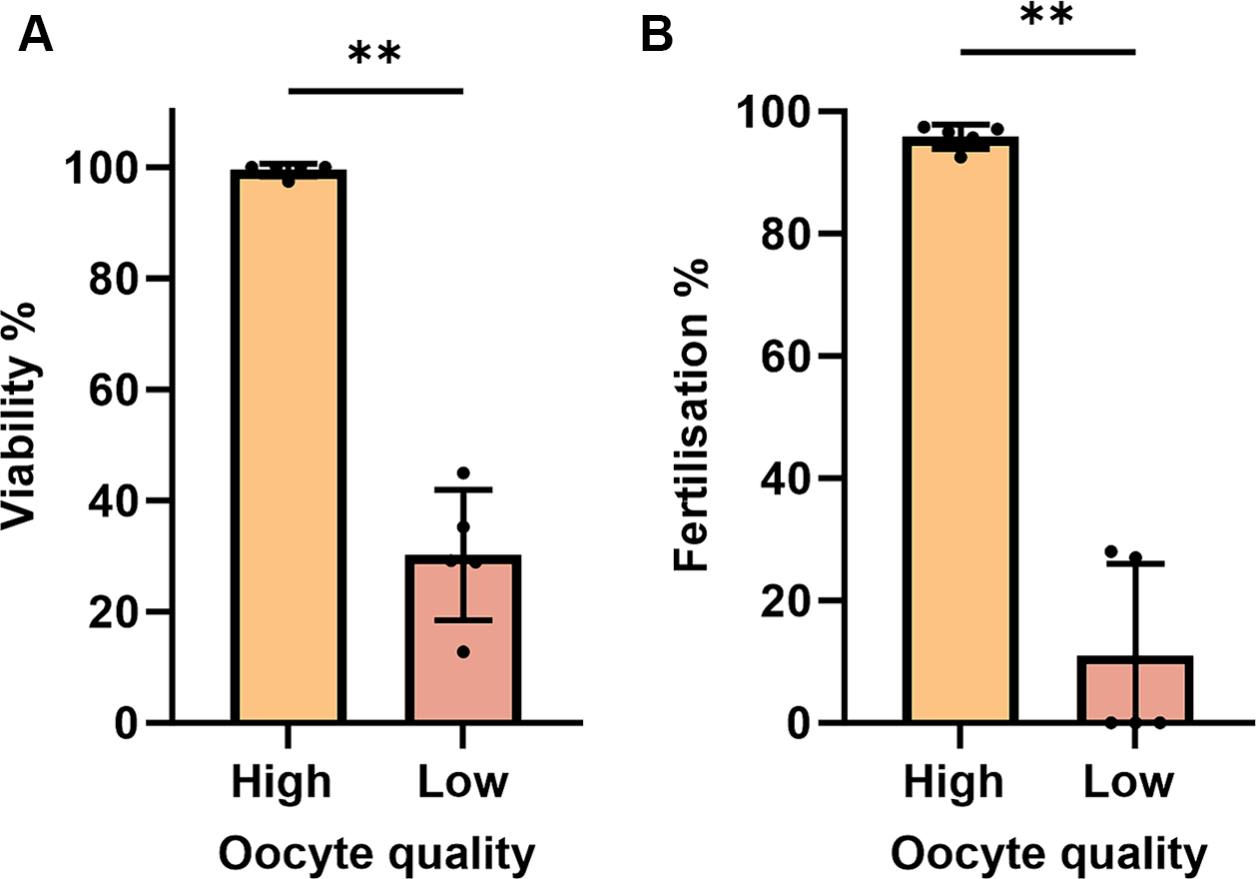
Evaluation of TB-based viability **(A)** and fertilization rates **(B)** during post-ovulatory aging in zebrafish. Data represents mean ± SD. Statistical significance calculated by one-way ANOVA followed by Tukeys multiple comparison test are shown by ns, non-significant; *p ≤ 0.05; **p < 0.01; ***p < 0.001; ****p < 0.0001.

**Figure 2:**
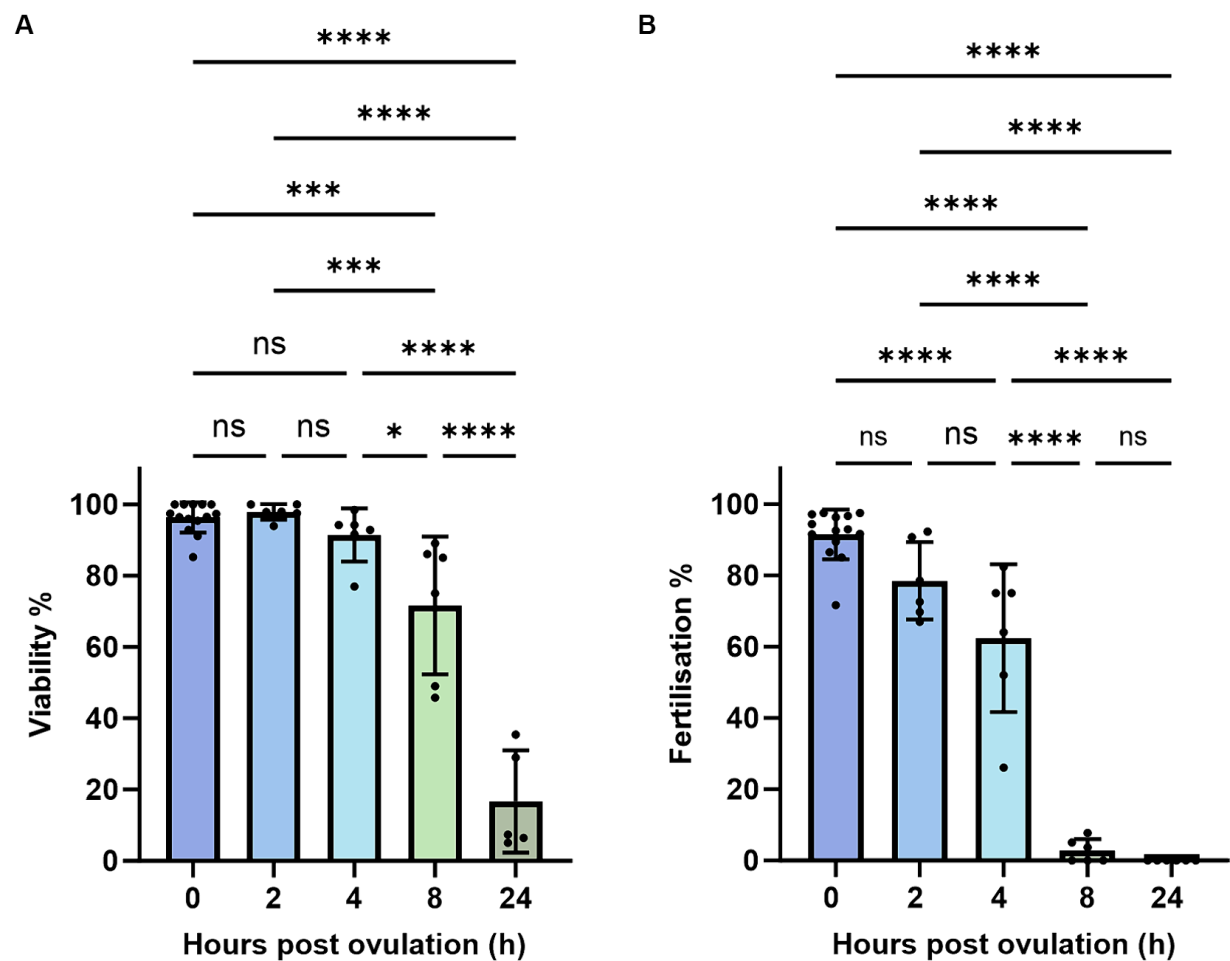
Oocyte quality of zebrafish assessed by TB-based viability **(A)** and fertilization rates **(B)**. Data represents mean ± SD. Statistical significance calculated by Mann Whitney test are shown by *p ≤ 0.05; **p < 0.01.

To account for quality eggs, we estimated oocyte viability by measuring the TB-based staining rate. An oocyte batch from a female with less than 50% viability rate at 0 HPO was considered poor and excluded from the study at further time points (Figure 1A). Those oocytes also exhibited very low fertilization rates of 27% at 0 HPO and were categorized as low-quality oocytes (Figure 1B). We observed hardly any impact during the first 4 h of *in vivo* oocyte aging with approximately 97% viability at 0 and 2 HPO, and 90% at 4 HPO without differences compared to 0 HPO (Figure 2A). The viability rate significantly decreased to 72% at 8 HPO and drastically to 16% at 24 HPO. The fertilization rate for oocytes fertilized immediately after ovulation was 91% and slightly decreased at 2 HPO to 78%, not reaching statistical significance (Figure 2B). However, this decrease significantly intensified at 4 HPO and 8 HPO to 62 and 2%, respectively. As expected, the fertilization rate at 24 HPO was nil.

### 3.2 Egg developmental indices

**Figure 3:**
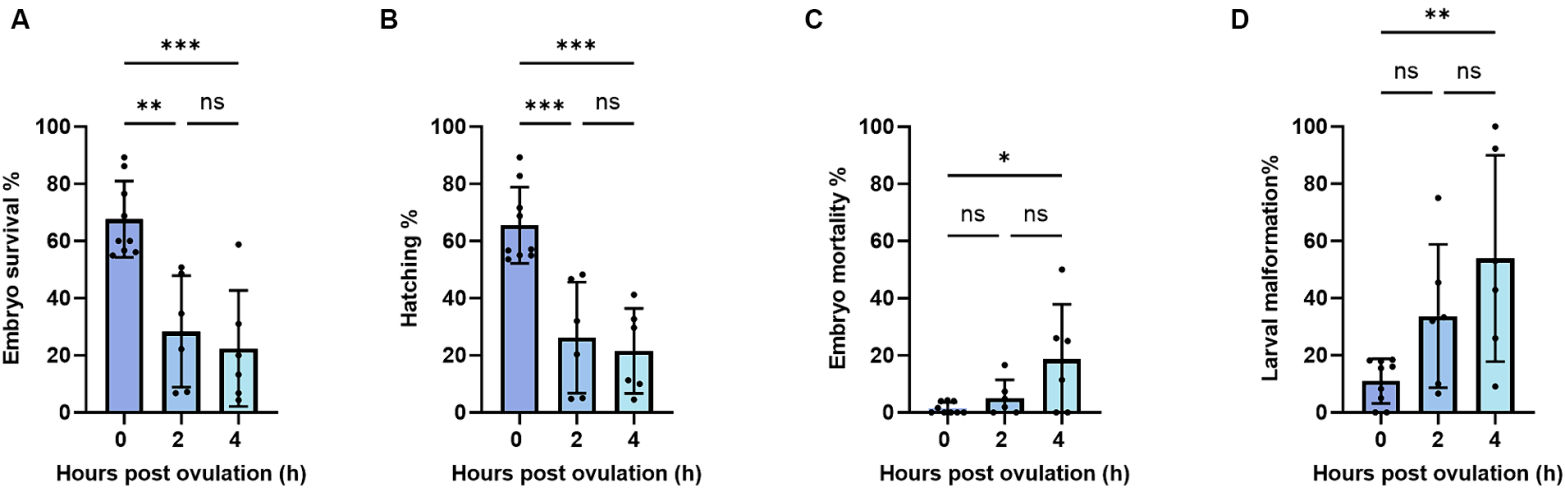
Embryo survival **(A)**, embryo mortality **(B)**, hatching **(C)**, and larval malformation **(D)** rates during post-ovulatory aging in zebrafish. Data represents mean ± SD. Statistical significance calculated by one-way ANOVA followed by Tukeys multiple comparison tests is shown by ns, non-significant; *p ≤ 0.05; **p < 0.01; ***p < 0.001; ****p < 0.0001.

To further analyze the impact of post-ovulatory aging on oocytes, we measured the survival and hatching of the fertilized eggs. We found that within 2 h of oocyte aging, embryo survival, and hatching rates had significantly declined from 67 and 65% at 0 HPO to 28 and 26% at 2 HPO, respectively. We did not reveal a further drop toward 4 HPO, which showed values of 27 and 22% (Figures 3A,B). Beyond that, the developmental indices are only shown up to 4 HPO, as no embryos developed from 8 HPO. Embryo mortality and larval malformation rates were assessed to further specify oocyte quality. Therefore, larva morphology was examined and classified as malformed if a curled, curved, or bent body shape was observed. The embryo mortality and larval malformation rates barely occurred at 0 HPO with 1 and 9%, respectively (Figures 3C,D). Although the mortality and malformation rates slightly rose to 5 and 33%, respectively, we did not detect significant differences for 2 HPO in either parameter (Figures 3C,D). However, at 4 HPO, a significant increase in the mortality and malformation rates to 12 and 41% was found (Figures 3C,D).

### 3.3 mRNA abundance of apoptosis-related genes in post-ovulatory aged oocytes

**Figure 4:**
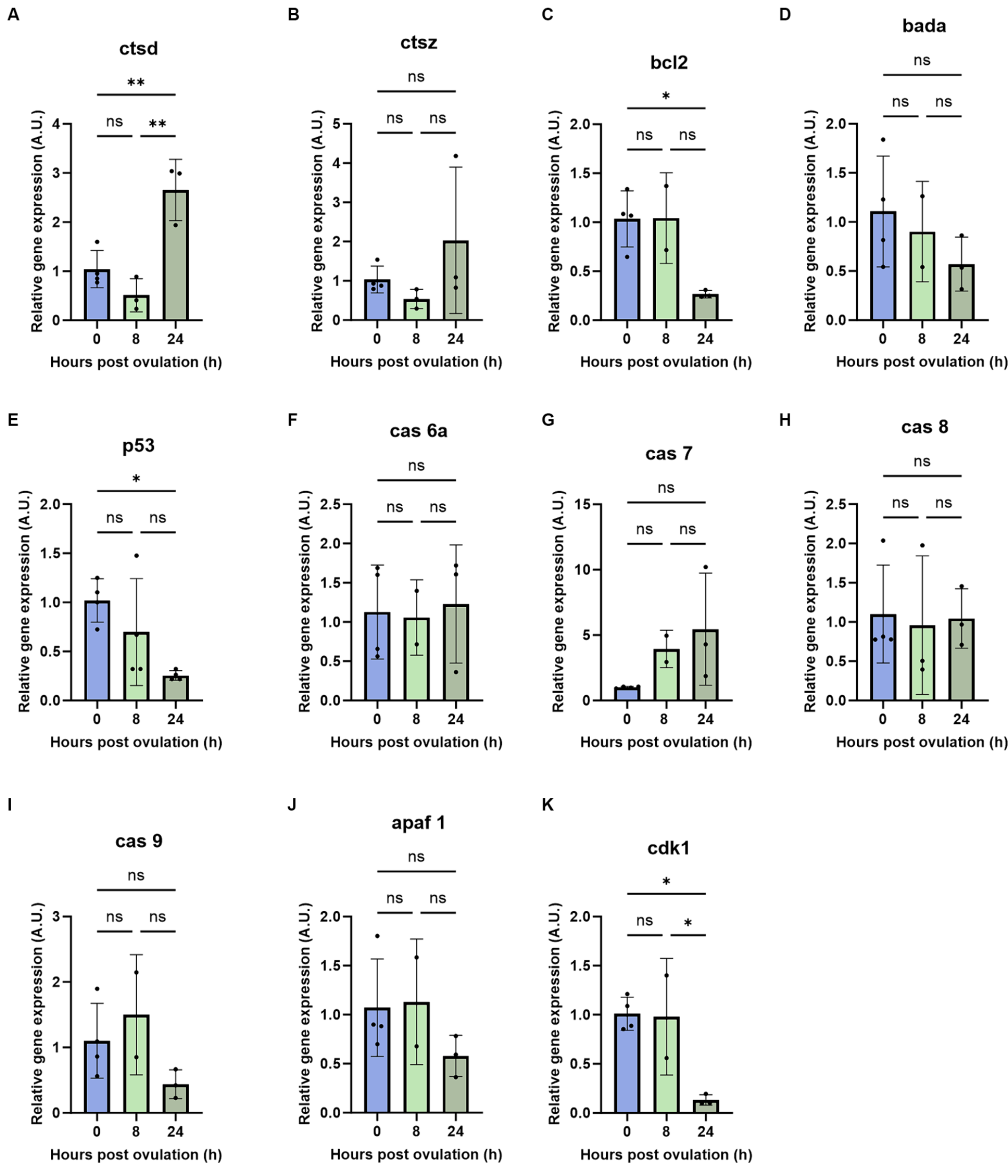
Regulation of pro- and anti-apoptotic genes during post-ovulatory oocyte aging. Depicted are the pro- and anti-apoptotic genes *cathepsin D* **(A)**, *cathepsin Z* **(B)**, *bcl2* **(C)**, bada **(D)**, *tp53* **(E)**, *caspase 6a* **(F)**, *caspase7* **(G)**, *caspase 8* **(H)**, *caspase 9* **(I)**, *apaf 1* **(J)** and *cdk1* **(K)**. A.U. represents arbitrary units. Data represents mean ± SD. Statistical significance calculated by one-way ANOVA followed by Tukeys multiple comparison test is shown by ns, non-significant; *p ≤ 0.05; **p < 0.01; ***p < 0.001; ****p < 0.0001.

To investigate possible mechanisms of post-ovulatory oocyte degradation, we studied the mRNA abundance of apoptosis-related genes during oocyte aging. Based on previous findings (17, 18, 33), mRNA abundance of pro- and anti-apoptotic genes were quantified at 0, 8, and 24 HPO. We observed a strong increase in the pro-apoptotic gene *cathepsin D* at 24 HPO compared to 0 and 8 HPO (Figure 4A). In line with the induction of apoptosis during oocyte aging, we also revealed a significant downregulation of the anti-apoptotic gene *bcl-2* at 24 HPO (Figure 4C). The pro-apoptotic gene *bada* showed constant transcript levels during all time points (Figure 4D). In contrast, the transcript level of *tp53* was significantly decreased at 8 and 24 HPO compared to 0 HPO (Figure 4E). The transcript level of the cell cycle-related gene *cdk1* significantly decreased at 24 HPO (Figure 4K). Furthermore, *cathepsin Z, caspase 6a, caspase 7, caspase 8, caspase 9*, and *apaf-1* enhibited no significant change at the examined time points (Figures 4B,F–J).

## 4 Discussion

The observed decrease in the fertilization rates during *in vivo* aging of zebrafish oocytes is consistent with findings in other species, such as rainbow trout, Japanese eel (*Anguilla japonica*), common carp, northern pike, and yellowtail tetra (*Alestopetersius caudalis*) (26, 34–37). However, the duration of oocyte storage assuring the fertilization success is relatively short in zebrafish compared to those other species. Zebrafish oocytes from certain females exhibited low TB-based viability rates at the time of ovulation, and hence, this decrease in viability was not solely attributed to aging. Various factors, such as nutrition, temperature, photoperiod, salinity, captivity management, stress, chemical elements, and diseases, can affect the oocyte quality (1) thus contributing to low viability rates at the time of ovulation. The *in vitro* aging of zebrafish AB strain oocytes at 26°C had no significant difference in fertilization rates until 2 h post-stripping, which aligns with our findings (31). However, it’s worth noting that the fertilization capacity of oocytes at 4 h of *in vivo* aging was higher, even at a higher storage temperature, compared to *in vitro* aging (31). Zebrafish Tubingen strain oocytes stored in Chinook Salmon ovarian fluid retained fertilizing ability for a longer duration (38) compared to our study. Conversely, gold-strain zebrafish oocytes stored at 8°C in a modified Hanks medium lost their fertilizing ability (39) earlier than *in vivo* storage of AB strain oocytes in our study. These variations in fertilization rates may be attributed to specific storage conditions (e.g., temperature and medium) as well as genetic strain differences. Although we observed high TB-based viability rates after 4 h *in vivo* aging, their fertilization rates significantly decreased. Hence, aging may cause the developmental capacity loss of oocytes at the early stage and viability loss in the later stage of aging.

The current results showed that the embryo mortality and larval malformation rates increased significantly with *in vivo* oocyte aging. However, it’s noteworthy that only 50% of the larvae were found to be malformed at 4 HPO, which contrasts with the findings from *in vitro* aging experiment where all larvae developed from 4-h-aged oocytes exhibited morphological malformations (31). Studies involving other fish species, such as northern pike, common carp, and rainbow trout, have consistently reported a higher occurrence of malformations in larvae originating from *in vitro* aged oocytes compared to those aged *in vivo* (17, 25, 26). This suggests that *in vitro* oocyte aging is more likely to result in malformation in the resulting larvae compared to *in vivo* aging. While it’s known that ovarian fluid components may contribute to maintaining the fertilizing ability and embryo development during oocyte aging (40), the precise role of ovarian fluid in the oocyte aging process remains elusive and requires further investigation.

The lysosomal proteases, including cathepsin D, cathepsin Z, and cathepsin B, can indirectly act as pro-apoptotic modulators (41). These cathepsins disrupt mitochondrial membrane potential, thereby triggering the mitochondrial pathway of apoptosis (42). In our study, we observed a significant increase in *cathepsin D* in 24-h-aged oocytes, while *cathepsin Z* showed a non-significant upward trend. Studies with other fish species have shown variability in cathepsin activity during oocyte aging. For example, sea bream (*Sparus aurata*) eggs with high cathepsin D activity exhibited activation of apoptosis (43), while mRNA abundance of *cathepsin D* showed no significant change during *in vivo* aging of rainbow trout oocytes (44). Similarly, studies with African catfish (*Clarias gariepinus*) and common carp showed varying trends in *cathepsin D* and *cathepsin Z* mRNA level (17, 33). Considering that matured oocytes are transcriptionally silent until the initiation of zygotic transcription and early embryo development relies on stored mRNAs (45), the increase in *cathepsin D* transcripts at 24 h post-ovulation in our study could be linked to the spontaneous activation of oocytes, which ceases transcriptional silence and presumably triggering cell death. Cathepsins are also known to potentially activate caspases, which are key regulators of apoptosis (46). In our study, we did not find a significant difference in the mRNA level of *caspase 6a*, *caspase 7*, *caspase 8*, and *caspase 9* in zebrafish aged oocytes. This contrasts with findings from studies involving common carp, where the mRNA abundance of *caspase 9* increased during oocyte aging (17). However, future research focusing on cathepsin and caspase enzyme activity may provide a clearer understanding of their roles and interactions during oocyte aging.

Another critical factor in cell fate determination is the ratio of pro-apoptotic to anti-apoptotic Bcl-2 family proteins. An excess of bcl-2 typically results in cell survival, while an excess of bax leads to cell death (47). In our study, we observed a significant decrease in *bcl-2* abundance in 24-h *in vivo* aged oocytes, which is consistent with findings in aged oocytes of pigs and mice (9, 10). However, the *bcl-2* transcript level remained unchanged in common carp aged oocytes during 14 h of *in vivo* aging (17). The *bax* gene is upregulated during oocyte aging in common carp (17), whereas it remains unchanged in mouse oocytes during *in vitro* aging (14). Additionally, in the current study, the expression of *bada*, which has a similar function to *bax*, showed no statistical difference during oocyte aging in zebrafish. This could be due to low levels of maturation promoting factor (MPF) and mitogen-activated protein kinase (MAPK) in aged oocytes (48) potentially influencing the downregulation of *bcl-2* without affecting *bada* expression (14). The decrease in the pro-survival gene *bcl-2* and the increase in pro-apoptotic *cathepsin D* might make aged oocytes prone to apoptosis.

p53can directly bind to PUMA/NOXA (49) and increase mitochondrial membrane permeability, resulting in leakage of pro-apoptotic proteins and cytochrome c, or indirectly activate pro-apoptotic genes, such as *bax*, *puma*, *bbc3*, *igfbp3*, *noxa*, and *apaf1* (50–52). The mRNA level of *tp53* decreased significantly at 24 HPO, which is consistent with the results observed in post-ovulatory aged rainbow trout oocytes (53). The transcript levels of tp53 orthologous decreased in maternally aged oocytes (54). The tp53 protein level decreased significantly in mouse oocytes (55), whereas the mRNA level remained constant in pig oocytes during post-ovulatory aging (56). Maternally derived *tp53* mRNA is essential for embryonic development in mice and frog (57, 58). Thus, a decrease in *tp53* transcripts along with *bcl-2* might contribute to abnormal cleavage, increased mortality, and increased malformations in embryos developed from the aged oocytes. However, the detailed role of *tp53* in oocyte developmental competence and aging is yet unexplored.

Cdk1 kinase is a major component of the maturation-promoting factor, MPF that maintains the ovulated oocytes at the metaphase arrest (59). Deficiency of *cdk1* in mouse ovulated oocytes prevents the cells from resuming the meiotic cycle and resulting in infertility (60). Post-ovulatory aged frog oocytes exhibited low cdk1 activity followed by spontaneous meiotic exit (61), which is a prerequisite for the activation of apoptosis (62). The drastic decrease in *cdk1* transcript at 24 HPO in our study might be due to the spontaneous activation to exit metaphase arrest and execute the cell death. In the current study, the 8-h-aged oocytes lost their fertilizing capacity and exhibited membrane blebbing phenotype after activation with water (unpublished data), which is a morphological indicator of apoptosis. Additional analyzes are required to elaborate on the mechanism behind spontaneous activation followed by cell death in post-ovulatory aged oocytes.

## 5 Conclusion

Zebrafish AB strain oocytes can be stored *in vivo* for 2 h with minimal loss of competency, and complete loss of egg fertilizing ability occurs at 8 h of ovulation. TB staining does not accurately detect oocyte post-ovulatory aging. However, it can be used as a simple and rapid method to estimate the quality of oocytes before fertilization in zebrafish. The upregulation of *cathepsin D* and downregulation of *bcl-2* and *cdk1* after 24 h of *in vivo* oocyte aging was observed. Based on the results obtained in the current study, the apoptotic pathway is activated in the advanced stage of aging, attributing to viability loss. Complementary analyses like DNA fragmentation, detection of proteins involved in the activation of cell death pathway, and examining mitochondrial changes would contribute to thoroughly understanding the involvement of apoptosis in the oocyte aging process.

## Data availability statement

The raw data supporting the conclusions of this article will be made available by the authors, without undue reservation.

## Ethics statement

The experiment was carried out under controlled conditions of RAS in the Laboratory of Intensive Aquaculture (LIA), which is part of the University of South Bohemia, Faculty of Fisheries and Protection of Waters (USB FFPW) (Vodňany, Czech Republic). All fish manipulations during the experiment were governed by valid legislative regulations of the Czech Republic (Act No. 166/1996 and No. 246/1992); the permit was issued No. 58672/2020-MZE-18134 and No. 33446/2020-MZE-18134 in the NAZV QK22020144 project.

## Author contributions

EK: Conceptualization, Formal analysis, Investigation, Methodology, Writing – original draft. KM: Writing – review & editing. SB: Writing – review & editing. SW: Formal analysis, Investigation, Methodology, Writing – review & editing. AAS: Formal analysis, Investigation, Methodology, Writing – review & editing. TP: Funding acquisition, Writing – review & editing. AIS: Conceptualization, Formal analysis, Funding acquisition, Investigation, Methodology, Supervision, Writing – review & editing.
