# Supplementary material for: Evaluation of viability, developmental competence, and apoptosis-related transcripts during in vivo post-ovulatory oocyte aging in zebrafish Danio rerio (Hamilton, 1822)
Source: Front Vet Sci. 2024 Jun 17;11:1389070. doi: 10.3389/fvets.2024.1389070 (PMC11216024; doi:10.3389/fvets.2024.1389070)

Supplementary data 1

Viability assessment of zebrafish oocytes at (A) 0 HPO, (B) 2 HPO, (C) 4 HPO, (D) 8 HPO, (E) 24 HPO using trypan blue staining technique. Scale = 2mm (A-E).

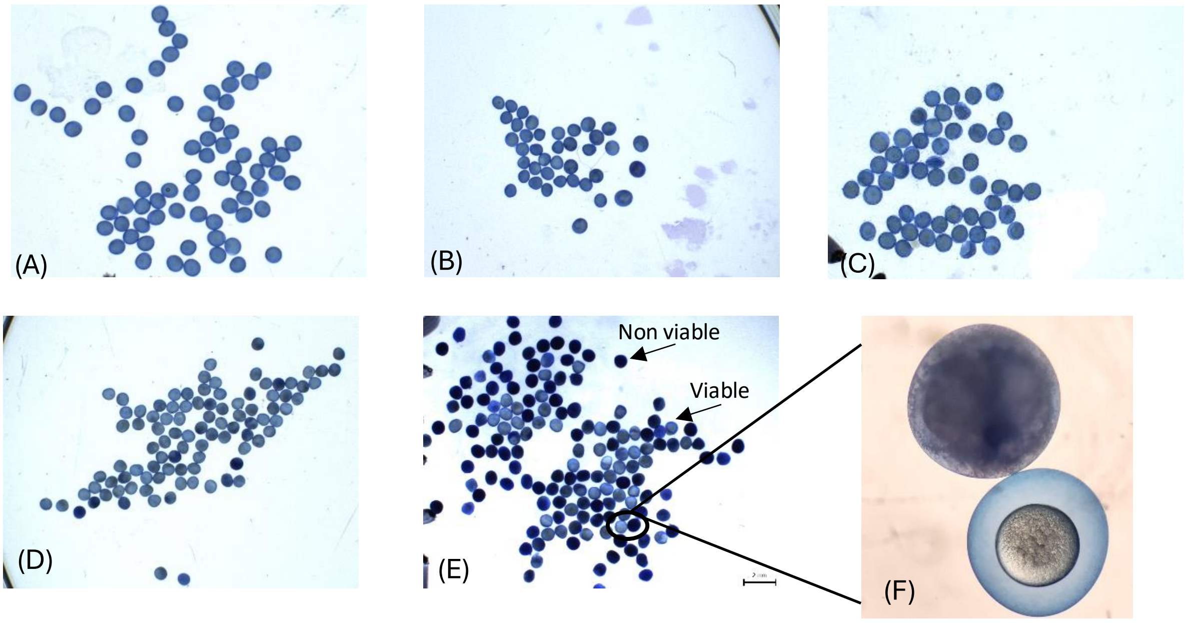

Supplement: Supplementary file 1 [file Image_1.pdf]
